# Development and Validation of Tumor Immunogenicity Based Gene Signature for Skin Cancer Risk Stratification

**DOI:** 10.3390/ijms222112025

**Published:** 2021-11-06

**Authors:** Maryam Yavartanoo, Gwan-Su Yi

**Affiliations:** Department of Bio and Brain Engineering, Korea Advanced Institute of Science and Technology (KAIST), 291 Daehak-ro, Yuseong-gu, Daejeon 34141, Korea; myavartanoo@kaist.ac.kr

**Keywords:** immunotherapy, clinical outcomes, prognosis, immune evasion

## Abstract

Melanoma is one of the most aggressive types of skin cancer, with significant heterogeneity in overall survival. Currently, tumor-node-metastasis (TNM) staging is insufficient to provide accurate survival prediction and appropriate treatment decision making for several types of tumors, such as those in melanoma patients. Therefore, the identification of more reliable prognosis biomarkers is urgently essential. Recent studies have shown that low immune cells infiltration is significantly associated with unfavorable clinical outcome in melanoma patients. Here we constructed a prognostic-related gene signature for melanoma risk stratification by quantifying the levels of several cancer hallmarks and identify the Wnt/β-catenin activation pathway as a primary risk factor for low tumor immunity. A series of bioinformatics and statistical methods were combined and applied to construct a Wnt-immune-related prognosis gene signature. With this gene signature, we computed risk scores for individual patients that can predict overall survival. To evaluate the robustness of the result, we validated the signature in multiple independent GEO datasets. Finally, an overall survival-related nomogram was established based on the gene signature and clinicopathological features. The Wnt-immune-related prognostic risk score could better predict overall survival compared with standard clinicopathological features. Our results provide a comprehensive map of the oncogene-immune-related gene signature that can serve as valuable biomarkers for better clinical decision making.

## 1. Introduction

Melanoma is considered a highly aggressive type of skin cancer, and the incidence rate of this type of malignancy has significantly been increasing over the past few decades [1]. Although melanoma accounts for less than 5% of all skin cancer types, it is responsible for approximately 75% of skin cancer deaths [2]. Metastasis in the brain is a common issue and accounts for the high death rate in patients with advanced melanoma [3]. Nearly 20% of patients diagnosed with melanoma are found to have brain metastasis at the time of diagnosis, which is significantly associated with poor prognosis and survival [3,4,5]. The survival rate dramatically decreases to 23% in metastasis patients [6]. The effort of most research has been focused on identifying reliable markers to evaluate and estimate the prognosis of melanoma patients [7,8,9,10]. The tumor microenvironment harbors cellular and non-cellular components. Cellular components such as immune cells have a strong influence on tumorigenesis, progression, and metastasis. Understating tumor immunity and the tumor microenvironment (TIME) led to the development of cancer immunotherapies. There are several extra and intracellular mechanisms correlated with tumor immunogenicity and immune escape. Immune escape has been considered a critical hallmark of solid tumors. Tumor cells may escape immune recognition through the downregulation of MHC class I expression [11], production of immunosuppressive cytokines such as TGF beta [12], IL10, and immune checkpoint proteins expressed on the cancer cell such as PDL1, CTLA-4, and TIGIT [13]. The activation of several oncogenes, such as Wnt/β catenin [13] and MYC [14], or loss of PTEN [15] function can trigger the production of immune suppression molecules. An extracellular mechanism such as hypoxia [16] or aberrant tumor vascularization [17] also has a high impact on tumor immunogenicity. Cancer immunotherapy strategies, including PD-1/PD-L1 and CTLA-4 inhibitors, have become one of the most important therapy for melanoma [18]. However, due to the complexity of the tumor microenvironment, only a minority of patients respond to it, and the majority have a partial response or no response to the therapy [19]. The discovery of biomarkers using public databases has been applied to investigate the prognostic markers in several cancer types. Currently, only a few prognosis models based on immune-related genes that systematically evaluate the tumor immune microenvironment (TIME) and predict the overall survival of melanoma patients are available [20]. Therefore, it is crucial to construct a prognosis gene signature that can provide better values for recognizing high-risk patients than the traditional tumor-node-metastasis (TNM) staging system, especially for the earlier stages. Here we aimed to discover novel biomarkers that would effectively predict low tumor immunity and the overall survival of melanoma patients. In the present study, a large cohort of melanoma patients from the Cancer Genome Atlas (TCGA) database was used to screen for the primary risk factor for low tumor immune-cells infiltration. Next, a prognosis gene signature was constructed. After constructing the risk signature score, multiple melanoma transcriptome datasets from the Gene Expression Omnibus (GEO) database were adopted as the validation sets for evaluating the result. Furthermore, the immune-related gene signature was analyzed in order to predict the durable clinical benefit of immune checkpoint blockade therapy using the melanoma immunotherapy dataset.

## 2. Results

### 2.1. Identification and Validation of Melanoma Immune Subtypes

**Figure 1:**
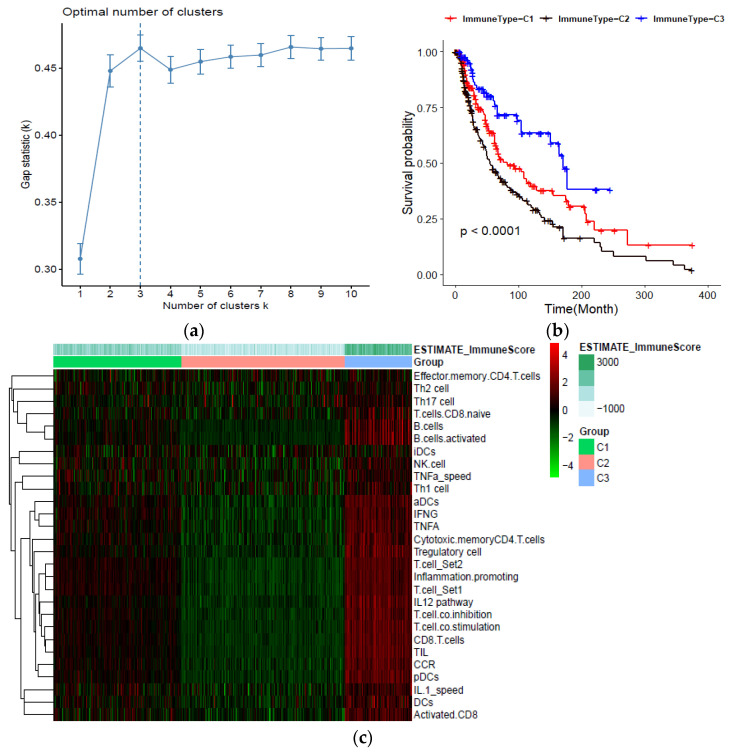
Classification of human skin cutaneous melanoma (SKCM) based on immune cells infiltration; (**a**) Estimation of optimal cluster number by gap statistics analysis; (**b**) Kaplan–Meier plot of overall survival for patients in the three immune-related groups; (**c**) Heatmap of the immune subtypes based on the ssGSEA scores for 28 immune gene sets.

The detailed workflow of the study design and analysis is shown in Figure S1. After data processing, a total of 461 TCGA–SCKM patients with an overall survival greater than 30 days were included for the rest of this study. The expression signature score of 28 immune gene-sets was used as the definitive input for the ssGSEA analysis and consensus clustering. Based on the comparison of the cumulative distribution function (CDF) curve from two to nine cluster numbers, the consensus clustering matrixes (Figure S2a,b), and the number of tests supporting the cluster number from the gap statistics analysis (Figure 1a), three distinct clusters were identified. PCA and the silhouette coefficient confirm the stability of the three clusters (Figure S2c,d). Patients in cluster C3 (*n* = 86) presented significantly better prognostic values for overall survival (OS), while patients in cluster C2 displayed a strong tendency for poor clinical outcomes (Figure 1b). The extent of the immune infiltration score increased in the following order: C3 > C1 > C2. Meanwhile, the ESTIMATE algorithm (a known immune scoring algorithm) was used to calculate the immune score of the three subtypes. For instance, C3 had the highest ssGSEA scores for the genes related to the Th1 cells, B cell, activated CD8 T cells, and immune checkpoint, along with the higher ESTIMATE immune score (Figure 1c). Then, the expression of seven immune checkpoint proteins (CD80, PDCD1, CD274, PDCD1LG2, CTLA4, HAVCR2, and LAG3) was analyzed. With respect to three immune subtypes, the expression of the checkpoint genes decreased in the sequence of C3 > C1 > C2 (Supplementary Figure S3a–g).

### 2.2. Wnt/β Activation Is Identified as the Primary Risk Factor for Low Immune Infiltration in Melanoma

**Figure 2:**
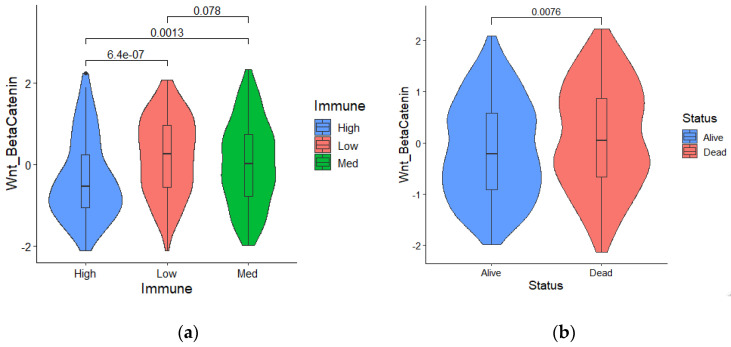
(**a**) Wnt/β catenin activation was significantly elevated in patients with low immune infiltration; (**b**) Survival status and a higher Wnt/β activation score are significantly correlated.

A literature survey was carried out to collect mechanisms and related gene signatures correlated with low tumor immunity or immune evasion. Based on the ssGSEA scores of cancer immune evasion mechanisms and immune subtypes information in the training set, the odds ratio of each mechanism was calculated and ranked. Compared with other cancer-immune-evasion mechanisms, such as Hypoxia, MYC activation, angiogenesis, and fatty acid metabolism, multinomial logistic regression analysis indicated that Wnt/β catenin activation was the primary risk factor among various hallmarks of cancer (odd ratio: 2.678, *p*-value < 0.01). Table 1 shows the odds ratio derived from multinomial regression analysis for each cancer-immune-evasion mechanism. Figure 2a shows that the Z-scores of the Wnt/β ssGSEA were significantly elevated in the low immune subtype compared with patients with higher immune cell infiltration. In addition, we found a negative correlation between increasing Wnt/β scores and survival status (Figure 2b).

### 2.3. Establishment of Prognosis Wnt–Immune–Related Gene Signature

**Figure 3:**
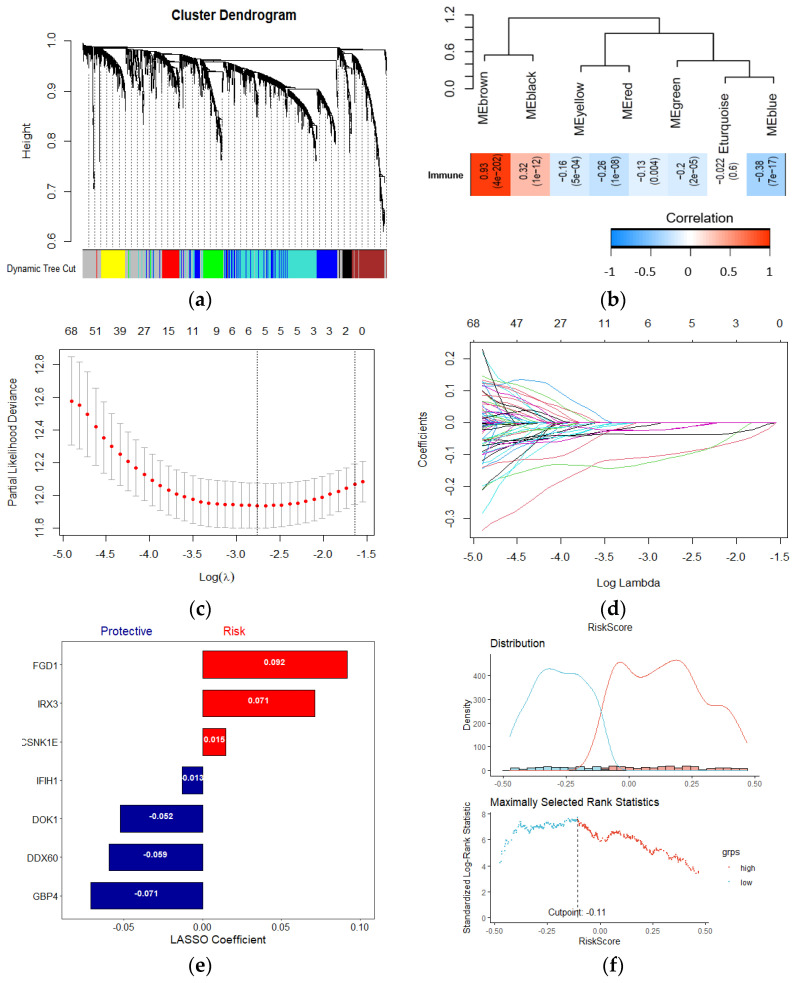
Establishment of a Wnt−immune-related gene signature. (**a**) WGCNA was performed with WNT beta catenin activation-related genes and immune scores; (**b**) A total of 7 non-grey modules were identified. The brown module, which shows the highest correlation (r = 0.93, *p* = 4e−202), was considered the most correlated with hypoxia; (**c**,**d**) The LASSO Cox regression model was used to identify the most robust markers, with an optimal λ value of 0.0515; (**e**) Distribution of LASSO coefficients of the WNT-Immune-related gene signature; (**f**) Scatter plot shows the standardized log-rank statistic value for each corresponding risk score cutoff.

To identify Wnt/β-related gene modules associated with the immune score, WGCNA was performed in the TCAG melanoma samples using known Wnt/β-catenin activation genes and immune scores (Figure 3a). In the presented study, the power of β = 3 (scale-free R^2^ = 0.95) was selected as the soft-thresholding parameter to construct a scale-free network (Figure S4a,b). In total, seven non-grey modules were identified. For genes that are not assigned to any of the modules and not co-expressed, WGCNA represents them in a grey module. Among all, the brown module showed the highest correlation with immune score (Figure 3b). The correlation between individual genes and biological traits (immune score) was defined as the gene significance (GS). Using the threshold of the *p*-value of <0.0001, 215 hub genes were extracted. The hub genes were submitted to the univariate cox regression analysis. With a threshold of *p*-value < 0.001, 70 genes significantly correlated with survival were identified. Then, the LASSO regression model, with an optimal lambda value of 0.0515, was used to find the most robust survival-related prognosis genes (Figure 3c,d). Here, in order to prevent overfitting, we used 10–fold cross–validation. Seven non-zero coefficient genes (IRX3, GBP4, CSNK1E, DOK1, FGD1, IFIH1, DDX60) were identified as final prognosis−related genes. The distribution of LASSO coefficients of the candidate genes is summarized in Figure 3e. Using the expression of each candidate gene and coefficient value derived from LASSO regression, the Wnt-immune risk score for each patient was constructed using the following equation: ∑i: Coefficient (*mRNAi*) × Expression (*mRNAi*). Finally, the estimation of the cutoff value for defining high- and low-risk score subtypes (Figure 3e) was performed by the maximally selected rank statistics method (the most significant split based on the standardized log-rank test), and patients were grouped into the low- and high-risk subtypes.

### 2.4. Wnt-Immune Risk Score Serves as a Risk Factor for Overall Survival Prediction

**Figure 4:**
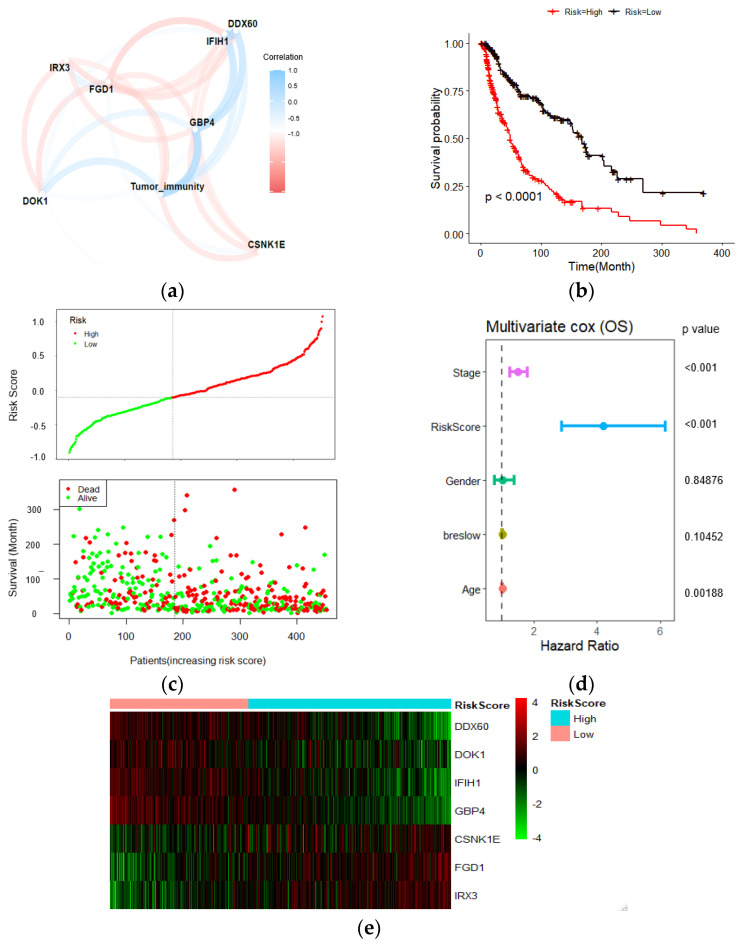
Prognostic analyses of 7 gene signatures in TCGA dataset. (**a**) Pearson correlations of the seven gene signatures with the tumor immune score. Four genes showed a positive correlation with tumor immune score, and three negatively correlated. The line thickness represents the correlation value; (**b**) Kaplan–Meier analysis showed higher risk scores correlated with worse OS; (**c**) Risk score distribution in TCGA dataset; (**d**) Multivariate Cox regression analysis revealed that risk score was an independent risk factor for OS; (**e**) Heatmap of the candidate genes in the prognostic classifier.

In the melanoma TCGA dataset, out of seven risk markers, four were shown to be positively correlated with the immune score, while three showed a negative correlation (Figure 4a). A Kaplan–Meier analysis showed that the patients with low-risk scores exhibited a better prognosis than higher risk-scored patients (Figure 4b). The risk curve and scatter plot illustrate that an increasing risk score was correlated with higher mortality (Figure 4c). In addition, among several clinicopathological features, the risk score driven from our candidate gene signature acted as an independent risk factor for overall survival (HR:4.2, *p*-value < 0.001) in the multivariate Cox regression analysis (Figure 4d). The heatmap of the risk score signature in this cohort showed that the expression levels of DDX060, GBP4, IFIH1, and DOK1 were higher in the low-risk group than in the high-risk group. In contrast, FGD1, IRX3, and CSNK1E expression were higher in the high-risk patients (Figure 4e).

### 2.5. Validation of Risk Score in the Test Datasets

**Figure 5:**
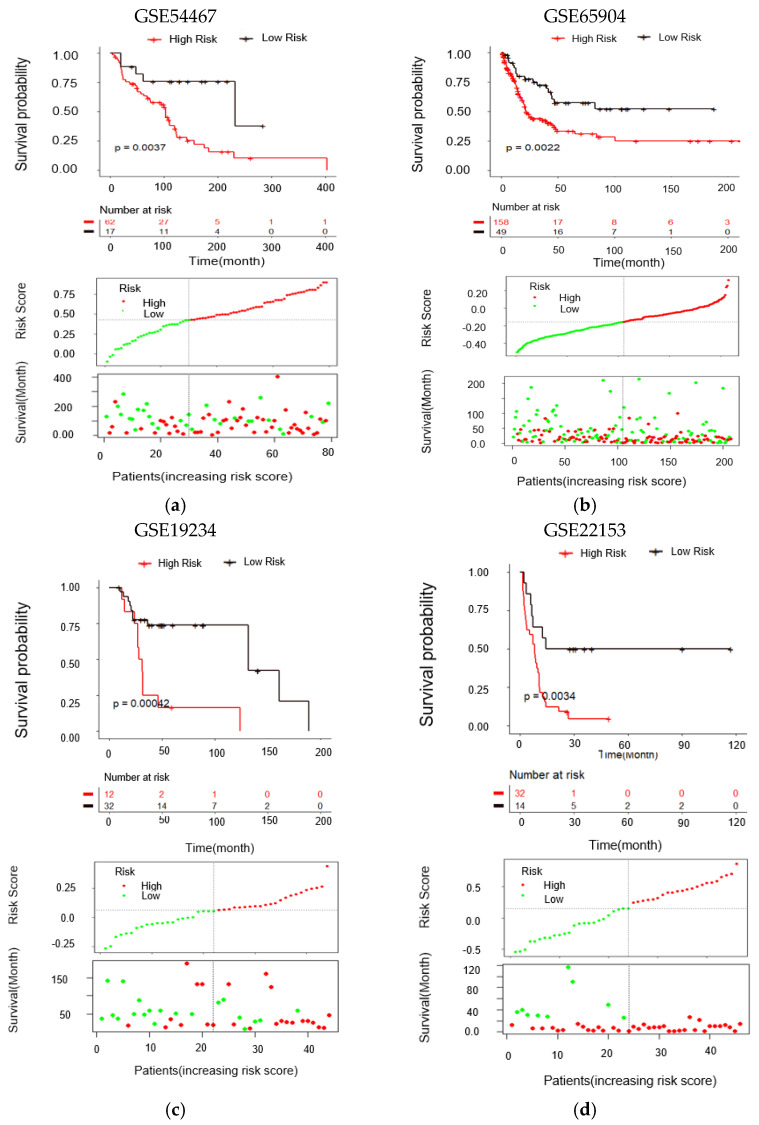
Validation of the gene signature in different melanoma transcriptome datasets. (**a**–**d**) Kaplan–Meier analysis, distribution of risk score of validation datasets I to IV, respectively. The black dotted line indicates optimum cutoff dividing patients into low-risk and high-risk subtypes. Statistical significance was determined by the log-rank test.

To confirm the robustness of the candidate biomarkers for predicting the survival of melanoma patients, the result was further validated in the four independent melanoma gene expression datasets. Similar to the training dataset, Kaplan–Meier analysis and risk score distribution revealed that higher risk scores predicted worse overall survival in all test datasets (Figure 5a–d).

### 2.6. Enriched Pathways, Immune Infiltration, and Genomic Alterations Analyses between Different Risk Groups

**Figure 6:**
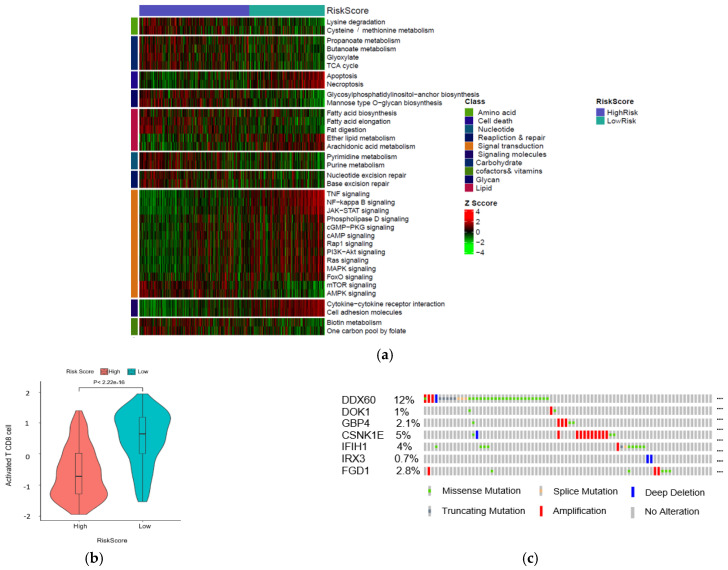

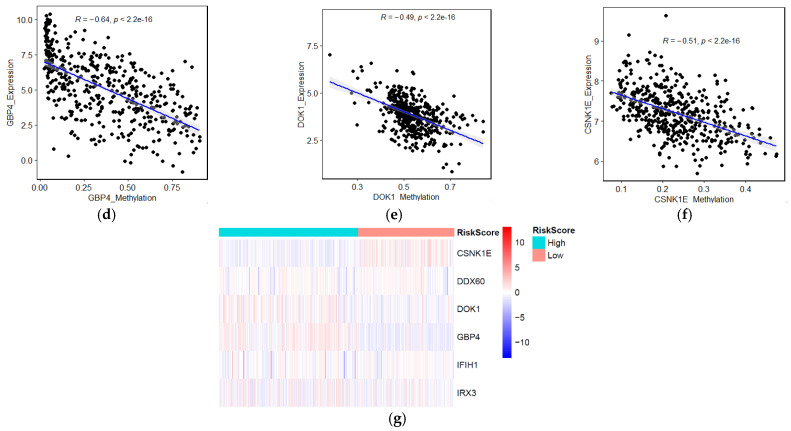
Identification of aberrant mechanisms in high-risk groups. (**a**) Heatmap shows normalized pathway enrichment scores in two high- and low-risk score subtypes. The 36 significantly enriched pathways are plotted (Fold changes >1.5, FDR < 0.01); (**b**) Boxplot of T cell infiltration score; (**c**) Frequency of the 7 prognostic genes in total samples from the TCGA dataset (Data were derived from the cBioPortal database). Correlations between mRNAs levels and methylation levels of GBP4 (**d**), DOK1 (**e**), CSNK1E (**f**); (**g**) Heatmap of methylation levels of candidate biomarker genes.

To further discover differences in pathways activation between high- and low-risk score patient groups, a single sample gene set enrichment analysis was performed using 10 KEGG pathways correlated with metabolism, environmental signaling, cell growth, and death. All pathways and correlated categories have been summarized in Table S3. In total, 36 pathways (Figure 6a) were differentially enriched between two groups (|FC| > 1.2, FDR < 0.01). The result showed that patients with higher risk scores had decreased activation of cell death-related pathways. In contrast, the mTOR signal transduction pathway was enriched in the high-risk group (Figure S5a,b). Next, we evaluate the correlation between risk score and T cell infiltration using the T cell score dried from the immune scoring step. As we expected, the result indicated that high-risk patients have lower CD8 T cells infiltration (Figure 6b). We further analyzed the occurrence of somatic mutations and their influences on gene expression. The top 20 most frequently mutated genes in each risk score group are shown in Figure S5c,d. By analyzing the TCGA genomics, we also found that the gene alteration rates in the melanoma TCGA dataset were 12% for DDX60, 5% for CSNK1E, 2.8% for FGD1, 2.1% for GBP4, 4% for IFIH1, 1% for DOK1, and 0.7% for IRX3 (Figure 6c), and these alterations were not significantly correlated with mRNA expression. Instead, we found the DNA methylation levels of protective genes such as GBP4, DOK1 negatively correlated with their mRNA levels (Figure 6d–f), suggesting that DNA hypermethylation may underlie the low expression of these genes in the high-risk group (low immunity). Moreover, hypermethylation of CSNK1E in the low-risk group (high-immunity) might be correlated with the lower expression of this gene. The heatmap summarized the gene signature methylation levels in high and low risk (Figure 6g). FGD1 was excluded from this analysis due to a lack of methylation data.

### 2.7. Wnt-Immune-Derived Signatures Predict Immunotherapy Response

**Figure 7:**
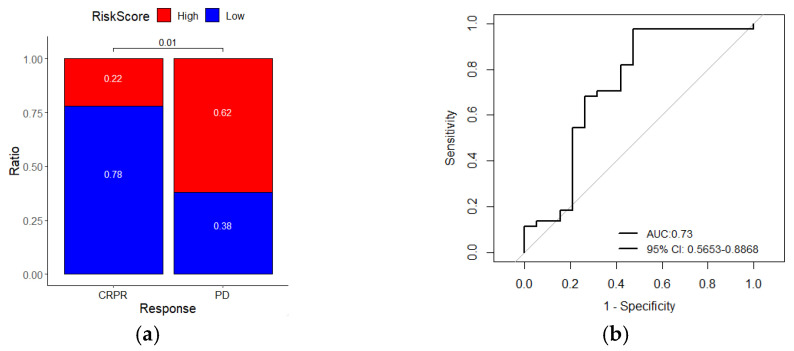

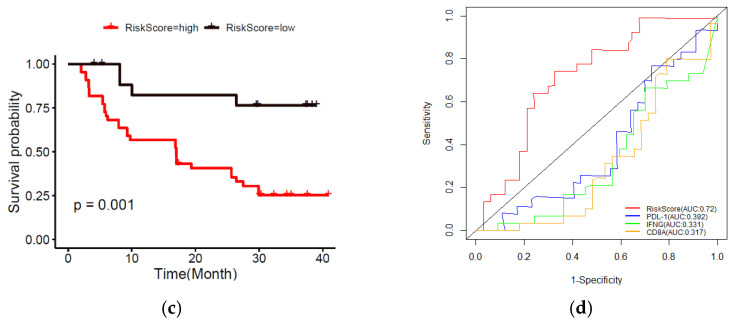
The risk score gene signature can predict the response of melanoma patients to anti-PDL1 therapy. (**a**) The ratio of immunotherapy responses of melanoma patients with high- or low-risk scores. Statistical significance was computed using the chi-square test; (**b**) ROC curves evaluate the accuracy of the signature for predicting the response of patients; (**c**) Kaplan–Meier curves of the overall survival of patients. Statistical significance was calculated using the log-rank test; (**d**) ROC curves of the sensitivity and specificity of the signature compared with other biomarkers in predicting the OS of patients in the immunotherapy dataset. CRPR, complete/partial remission; PD, progressive disease.

Next, we evaluated whether the signature could predict the response to immune checkpoint blockade (ICB). By analyzing a melanoma immunotherapy dataset (GSE91061), we found that patients with low-risk scores had a higher immunotherapy response rate than patients with high-risk scores (chi-square test, *p* = 0.01, Figure 7a). The ROC curve also showed that the gene signature could predict the Nivolumab therapy response of melanoma patients (AUC = 0.726, 95% CI = 0.5653–0.8868, Figure 7b). Kaplan–Meier analysis of this cohort showed that patients with high-risk scores had a worse survival rate than patients with low-risk scores (log-rank *p* = 0.001, Figure 7c). ROC curves for survival prediction showed that the risk score had a higher AUC than other immune-related biomarkers (PD-L1, CD8A, and IFNG, Figure 7d). In summary, these results suggest that the seven-gene signature is a potential predictor of immunotherapy response.

### 2.8. Construction and Validation of Nomogram Based on Immune-Related Gene Signature and Clinicopathological Risk Factor

**Figure 8:**
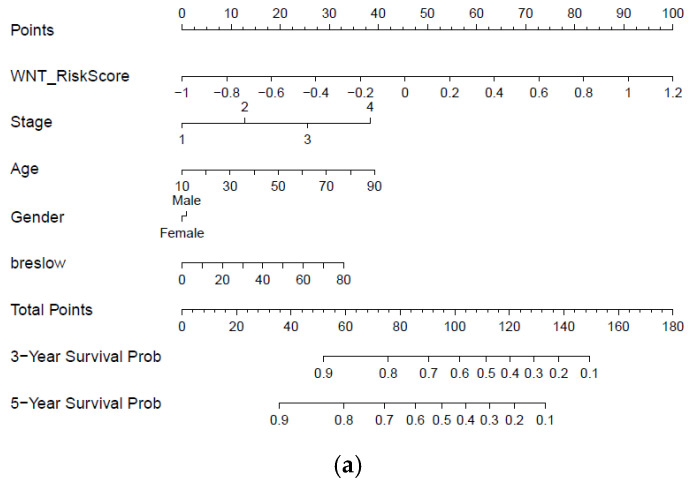

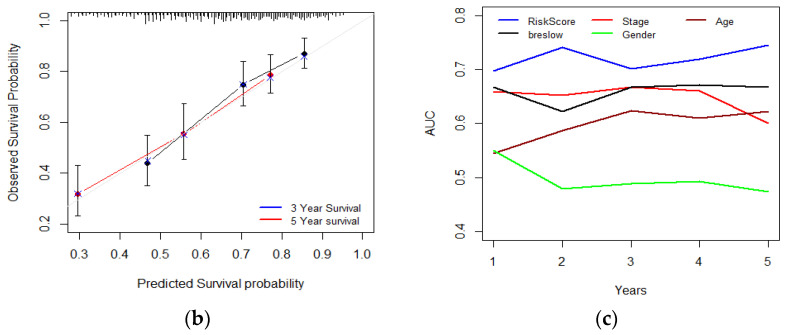
Developing and validating a nomogram based on the immune-related gene signature risk score model. (**a**) A prognostic nomogram created by integrating the risk score and clinical parameters; (**b**) The nomogram displays high accuracy in the calibration analysis; (**c**) AUC curves of risk score-related gene signature nomogram compared with stage, age, Breslow, and gender in the melanoma-TCGA (1 to 5 years).

Furthermore, with the integration of risk score, age, tumor stage, gender, and Breslow thickness, a nomogram using the TCGA melanoma dataset was established to predict individual risk of three- and five-year survival (Figure 8a). The result indicates that the seven-gene signature is significantly associated with the clinical outcome of melanoma patients. Compared with the ideal model (45-degree grey line), the calibration plots for three and five-year survival rates were predicted well in the TCGA dataset Figure 8b. The result of the ROC analysis revealed that the nomogram prediction efficiency was better than other clinicopathological features (Figure 8c). Finally, the nomogram result was validated in the independent dataset (Figure S6a,b). The validation result confirmed the robustness of the nomogram. In summary, the nomogram showed the most potent and stable ability for survival prediction, with an average AUC above 0.7, better than the pathological TNM staging alone.

## 3. Discussion

Melanoma is considered the most aggressive and fatal type of skin cancer, with the challenge of identifying reliable and robust prognostic biomarker candidates. As tumor immunogenicity is an important factor that may confound immunotherapy response and tumor progression, the development of immune-related biomarkers can provide a new approach to clinical design making and individualizing treatment. So far, some immune-related gene signatures have been established for survival prediction in different cancer types, including melanoma, bladder, and breast cancer. Several recent studies constructed their gene signatures by only comparing high and low immune infiltration subtypes using immune-related genes from previously published literature. Although, this type of study is commonly used, the underlying mechanism of tumor low immunity cannot be identified through the analysis processes. Understanding the relationships between immune infiltrations with cancer cells or environmental mediators empowers the characterizing of tumor complex biology, thereby supporting more precise therapy decisions. In this study, out of various tumor-related mechanisms, we identified Wnt/β activation as the primary risk factor for low immune infiltration using ssGSEA and multinomial logistic regression in the TCGA melanoma dataset. WGCNA was performed to identify Wnt/β activation-related gene modules based on transcriptome profiling data. Next, the univariate and LASSO Cox regression analyses were preformed to screen robust prognostic biomarkers and establish a Wnt-immune-related gene signature. Afterward, the prognostic value of the gene signature was validated in four independent melanoma transcriptome datasets. In all validation datasets, the gene signature showed the capacity to discriminate high-risk patients. The result suggested that the signature can serve as a reliable risk factor for melanoma patient stratification. Enriched pathways, genomic alterations were also analyzed and compared in different risk score groups, and we observed that a high-risk score was significantly correlated with more aggressive molecular changes, such as enriched mTOR activation, and downregulation of the apoptosis pathway. Interestingly, pathways correlated with amino acid and fatty acid metabolisms were differentially enriched between two risk score groups, suggesting a potential correlation between Wnt/β catenin activation and the metabolic reprogramming of cancer cells which can affect tumor immunogenicity and survival. Seven genes identified by this study can bring new insights into melanoma progression. In the current study, we found that higher expressions of FGD1, CSNK1E, and IRX3 are correlated with shorter survival of melanoma patients. Faciogenital Dysplasia 1 (FGD1) was involved in multiple biological processes, such as cell cycle progression and cell polarity, and exhibits oncogenic behaviors in hepatocellular carcinoma and osteosarcoma [21,22]. It has been known that FGD1 regulates the expression of PDL1 in a PTEN-dependent manner and regulates PDL1 therapy resistance [21]. CSNK1E (Casein Kinase 1 Epsilon) has been found as a synthetic lethal (SL) to TP53 and has been suggested as a promising target for TP53-mutated cancer patients [23]. This gene is a known component of the Wnt signaling pathway, and in response to WNT signaling, CSNK1E phosphorylates a large number of proteins [24]. Interestingly, we found that a higher expression of CSNK1E is correlated with shorter survival in melanoma patients. This gene might act as an SL pair for mutated TP53 in melanoma patients, which constitute ~30% of patients and, therefore, a potential novel therapeutic target in this type of tumor. IRX3 (Iroquois Homeobox 3) is a risk biomarker for melanoma patients. IRX3 has been suggested as a tumor-promoting gene in several tumors such as hepatocellular carcinoma [25] and may contribute to tumor angiogenesis [26]. Based on the literature, these three genes may have oncogenic properties in melanoma and can be considered novel melanoma therapeutic biomarkers. We also found that the overexpression of GBP4, DOK1, DDX60, and IFIH1 is correlated with better overall survival in several melanoma datasets. Guanylate-binding protein 4(GBP4) belongs to the interferon-stimulated factor that acts as a protective factor in host defense, and several genes from the GBP family act as tumor suppressors [27,28]. In addition, a recent study found the GBP4 was positively correlated with immune cell infiltration in melanoma patients [29]. Docking protein-1 (DOK1), a tumor suppressor, is frequently downregulated in human tumors such as ovarian cancer, Burkitt lymphoma, head and neck cancer (HNC), chronic lymphocytic leukemia (CLL), lung cancer, and breast cancer [30,31]. Based on our result, we hypothesize that this gene might act as a tumor suppressor in melanoma. DExD/H-Box Helicase 60 (DDX60) is involved in RIG-I-dependent and independent immune response [32]. Interferon induced with Helicase C Domain 1 (IFIH1) is a member of the IFN gamma family and has been suggested as an inducer of growth inhibition or apoptosis of multiple types of cancer cells [33]. Our result found a positive correlation between the expression of this gene and longer survival. A recent study emphasized the function of Wnt/β catenin activation in immunotherapy resistance [34]. Therefore, we analyzed the prediction power of the signature in immunotherapy response. In addition to the survival prediction power of the signature, we found that in the immunotherapy dataset, patients with higher risk scores exhibited less therapeutic responses compared to lower risk score patients, which indicates the gene signature could also serve as a potential marker of immunotherapy response prediction in melanoma. In the present study, by integrating several features, we generated a nomogram model. Nomograms combine multiple clinical features, and thus nomograms have become a powerful and easy-to-use tool to evaluate the survival probability of cancer patients. Notably, the results from this study showed that the immune-related gene signature nomogram had significantly higher efficiency than the staging system alone, with an average AUC above 0.7. The genes found by this study could serve as prognosis and disease progression biomarkers. The Wnt-immune-related signature constructed by this study could serve as prognosis, disease progression, and immunotherapy biomarkers.

## 4. Materials and Methods

### 4.1. Data Collection and Preprocessing

Gene expression data and clinical information of melanoma patients were downloaded from the Gene Expression Omnibus datasets (GEO) and The Cancer Genome Atlas (TCGA). For the microarray dataset (GSE54467, GSE19234, GSE19293, GSE22153, and GSE65904), background correction and normalization were performed by applying the robust multi-array averaging algorithm [35]. The TCGA read counts data were normalized using the TMM method implemented in the “*edgeR*” Bioconductor package [36] and then recalculated for a library size of one million. Finally, the calculated read counts per million (CPM) values were used as a measure of the mRNA level of a gene for the rest of the analysis. The RAN sequencing data of the malignant melanoma subjects that received anti-PD-1 and anti-CTLA4 therapy (GSE91016) was also downloaded from the GEO database. Patient 3 was excluded from the analysis due to the authors’ annotations [37]. Next, the raw count data was normalized and quantified by the “*edgeR*” package. All the gene expression levels have been log-transformed. Patients with an unknown diagnosis/follow-up date or survival status were excluded from this study. Clinical features for data are summarized in Table S1.

### 4.2. Identification and Validation of Melanoma Immune Subtypes

The immune score for each sample in the training set was calculated by determining the expression signature score for 28 immune gene-sets that have been reported as indicators of immune cell infiltration [38,39]. The gene sets include tumor immune-related cells such as T cell, B cell, and NK cell, and the gene set was correlated with activated immune cell products such as IFNG signaling pathways, IL1, and TNFA signaling pathway, etc. Table S2 summarizes all the immune gene sets and related references. We used the single-sample gene set enrichment analysis (ssGSEA) method implemented in the “*GSVA*” package (which calculates a gene set enrichment score per sample as the normalized difference in empirical cumulative distribution functions of gene expression ranks inside and outside the gene set) [40] to determine the enrichment score of 28 immune-associated gene sets in each human skin cutaneous melanoma (SKCM) sample in the TCGA database. Next, consensus clustering of patients based on their ssGSEA result was performed using the “*ConsensusClusterPlus*” package [41]. In summary, the k-mean clustering algorithm was used, with 1000 iterations and each using 80% of the overall samples. The optimal number of clusters was evaluated using the “*NbClust*” R package (version 3; http://cran.r-project.org/web/packages/NbClust/index.html (accessed on 5 November 2021)). In addition, the silhouette method and principal component analysis (PCA) were used to assess the optimal number of clusters. Next, the ssGSEA score *x_i_* for each TCGA melanoma patient *i* was rescaled to

$$x_{i}^{\prime }$$

using Equation (1).

(1) $$x_{i}^{\prime }=\frac{x_{i}-x_{min}}{x_{max}-x_{min}}$$

where *x_max_* and *x_min_* represent the maximum and minimum ssGSEA scores obtained from the melanoma TCGA dataset, respectively, then “*pheatmap*” package in R (version 1.0.12; https://CRAN.R-project.org/package=pheatmap (accessed on 5 November 2021)) was used for heatmap visualization of the clustering result. The ESTIMATE algorithm [42] was used to calculate the immune score in each subtype. The algorithms use gene expression data to infer the tumor cell composition and infiltration. The algorithm was implemented using the “*ESTIMATE*” package in R.

### 4.3. Candidate Biomarkers Selection and Signature Construction

First, the performances of several known cancer immune evasion mechanisms in the training set were quantified by a single-sample gene set enrichment analysis (ssGSEA) algorithm based on TCGA melanoma transcriptome profiling data and immune evasion mechanism-related gene sets from the Molecular Signatures Database (MSigDB) [43]. Table S3 summarized the mechanisms and related gene signatures. Multinomial logistic regression (MLR) analysis [44] using R package “*nnet*” [45] was performed to identify the effect of each mechanism on tumor immunity. Multinomial logistic regression is often used for modeling the association between covariates and the likelihood of observing a particular categorical outcome and can deal with categorical target variables with more than two classes [46]. A total of 1877 genes associated with aberrant Wnt/β-Catenin signaling from cell lines or tumor tissue-based studies were collected from the literature [47,48,49,50,51,52,53]. The Wnt/β activation-correlated genes were used to construct a scale-free co-expression network and identify an immune-related module (a set of co-expressed genes which highly correlated with the immune score) based on the patient’s transcriptome profiling data and immune scores. The R package “*WGCNA*” (weighted gene co-expression network analysis) was used for this analysis [54]. With a threshold of the *p*-value of GS <0.0001 and the *p*-value of univariate Cox regression <0.001, candidate genes from the ‘immune-related module were identified. Gene significance (GS) computes the association of individual genes with the immune score. Next, a least absolute shrinkage and selection operator (LASSO) Cox regression model was applied to screen for the most robust prognostic markers. The 3-fold cross-validation and 1000 iterations were performed to eliminate the potential instability of the result. The optimal tuning parameter λ was selected through the 1-SE (standard error) criteria. A Wnt-immune-related risk score for each patient was construed as follows:

(2) $$\mathrm{Risk}\;\mathrm{score}=\overset{n}{\underset{i=1}{\sum }}\left(Exp_{genei}\times \;\beta_{genei}\right)$$

where *βgene_i_* indicates the coefficients derived from the LASSO model and *Exp_genei_* represents the relative gene expression value. An optimal cutoff value was identified using the maximally selected rank statistics method. Subsequently, a Kaplan–Meier survival curve was constructed to evaluate the survival of the high- and low-risk group of patients. The result was validated using the validation GEO datasets.

### 4.4. Further Bioinformatics and Statistical Analyses

The KEGG pathways were downloaded from the Kyoto Encyclopedia of Genes and Genomes (KEGG) database 2021 (https://www.genome.jp/kegg/ (accessed on 5 November 2021)). A total of 169 human pathways were collected and clustered into 10 major categories based on the KEGG classification. Single set gene set enrichment analysis (ssGSEA) was utilized to compute the enrichment score of each pathway in each melanoma TCGA sample. In order to identify pathways that are differentially enriched among high- and low-risk score subtypes, the *“limma”* R package [55] was used. The Benjamini–Hochberg (BH) false discovery rate method was applied for p-value adjustment. Adjusted *p*-value less than 0.01 and FC > 1.5 were considered to be included for the rest of the analysis. The results were visualized by using the “*ComplexHeatmap*” R package [56]. The T cell infiltration gene–set from Table S2 was used to calculate the score of T cell infiltration for risk score subtypes. The correlation of each gene expression and methylation level score was calculated by the Spearman correlation. The correlation of the immune-related risk score with the response to immunotherapy was further investigated using the anti-PD1 RNA–seq dataset (GSE91061). All independent prognostic predictors, including age, tumor stage, and candidate signature scores, were applied to develop a prognostic nomogram. Receiver operating characteristic (ROC), area under the curve (AUC), and calibration curves were plotted to predict the discrimination and accuracy of the nomogram. Next, decision curve analysis (DCA) was performed to assess the clinical utility of the nomogram. Finally, the result was validated on the external GEO dataset. A nomogram and calibration curve were constructed using the R package “*rms*”.

### 4.5. Statistical Analysis

All analyses were performed using R software (version 4.1.0; https://www.r-project.org/ (accessed on 5 November 2021)) version 4.0.0 and corresponding packages. Kaplan–Meier analysis was further conducted to evaluate the relationship between immunogenicity score and overall survival using the “*survimer*” R package. The “*glmnet*” R package was used for LASSO analysis. Multiple testing was adjusted by the FDR method. In this study, FDR < 0.05 was considered a “significant” result.

## 5. Conclusions

In summary, we have successfully constructed a predictive model which combined the oncogene-immune-related genes signature with clinical characteristics to estimate the overall survival of melanoma patients. The candidate prognostic signatures developed by this study might serve as a prognostic classifier for clinical decision making and therapeutic strategies.

## Figures and Tables

**Table 1:** Result of multinomial logistic regression in melanoma TCGA sample.

Dependent Variable		
	Low Immune	High Immune
Hypoxia	2.459 *	0.556
Wnt/β catenin Signaling Pathway	2.678 **	0.718
TGF-β Signaling Pathway	1.749	0.274 **
DNA Repair	0.6	1.628
NOTCH Signaling Pathway	1.524	0.276 **
PI3K_AKT_MTOR Signaling Pathway	0.391 **	0.878
MTORC1 Signaling Pathway	0.779	1.485
MYC Oncogene activation	0.98	3.345 *
EMT	1.694	1.18
Fatty Acid Metabolism	2.143	0.097 ***
Oxidative Phosphorylation	0.442	4.502
Glycolysis	2.248	0.194 **
P53_Pathway	1.289	1.323
Angiogenesis	0.607	0.115 **
Mismatch Repair	1.067	0.625
MAPK Signaling Pathway	0.180 ***	3.115
Antigen Processing and Presentation	0.026 **	4.273 ***
*p*-value:	* <0.05; ** <0.01; *** <0.001	

Note: An odds ratio above 1 indicates that there is a higher likelihood of having the outcome, and an odds ratio of below 1 means that there is a smaller likelihood of having the outcome.

## Data Availability

The melanoma TCGA dataset analyzed by this study is available in The Cancer Genome Atlas repository, (TCGA; https://portal.gdc.cancer.gov). The GEO datasets analyzed here are available in the Gene Expression Omnibus repository, https://www.ncbi.nlm.nih.gov/geo/query/acc.cgi?acc=GSE54467, https://www.ncbi.nlm.nih.gov/geo/query/acc.cgi?acc=GSE91061, https://www.ncbi.nlm.nih.gov/geo/query/acc.cgi?acc=GSE65904, https://www.ncbi.nlm.nih.gov/geo/query/acc.cgi?acc=GSE19234, https://www.ncbi.nlm.nih.gov/geo/query/acc.cgi?acc=GSE22153 (accessed on 5 November 2021).

## Supplementary Materials

The following are available online at https://www.mdpi.com/article/10.3390/ijms222112025/s1.
