# Supplementary material for: Development and Validation of Tumor Immunogenicity Based Gene Signature for Skin Cancer Risk Stratification
Source: Int J Mol Sci. 2021 Nov 6;22(21):12025. doi: 10.3390/ijms222112025 (PMC8584987; doi:10.3390/ijms222112025)
Supplement: Supplementary file 1 [file ijms-22-12025-s001.zip › ijms-1433630-supplementary.pdf]

International Journal of *Molecular Sciences*

MDPI

Article

Development and Validation of Tumor Immunogenicity Based Gene Signature for Skin Cancer Risk Stratification

Maryam Yavartanoo[1], Gwan-Su Yi[1]*

[1] Department of Bio and Brain Engineering, Korea Advanced Institute of Science and Technology (KAIST), 291 Daehak-ro, Yuseong-gu, Daejeon, 34141, Republic of Korea; myavartanoo@kaist.ac.kr

* Correspondence: gwansuyi@kaist.ac.kr; Tel.: +82-42-350-4318

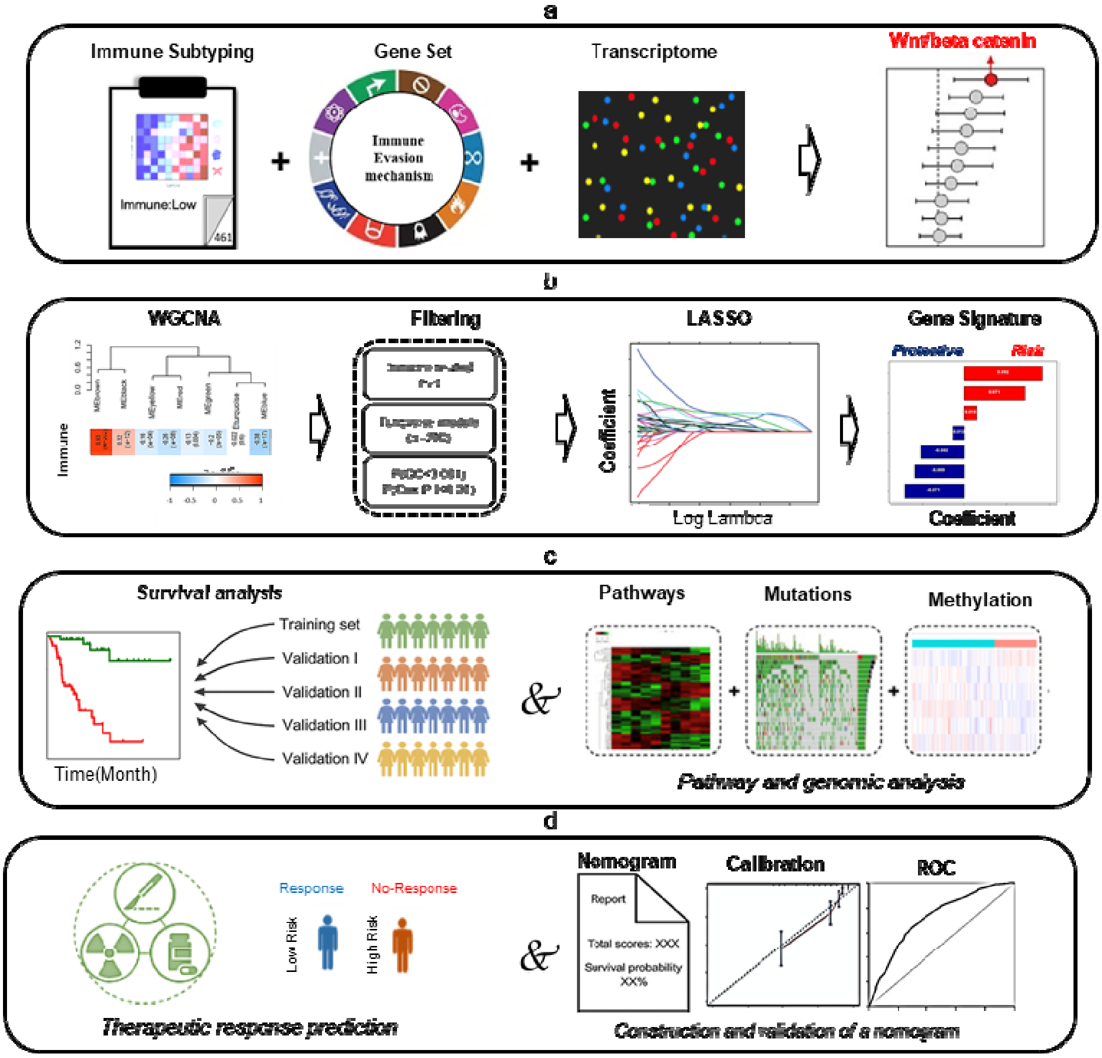

Figure S1. Flow chart indicating the process used to select prognostic immune-related genes included in the analysis

Table S1. Baseline characteristics of patients in the discovery and validation cohorts

GEO ID	Platform	No. of samples	Death event	Average OS (months)	Ages (years)	Gender (male/ female)	Primary/ Metastasis	Stage (I/II/III/IV)	Therapy response
GSE22153	GPL6102	53	44	14	53.5 ± 28.5	28/25	0/53	0/0/0/53	NA
GSE54467	GPL6884	79	49	97.93	51.5 ± 32.5	50/29	0/79	0/0/79/0	NA
GSE65904	GPL10558	214	102	33.18	62.35 ± 14.40	124/89	16/188	NA	NA
GSE19234	GPL570	38	24	56.65	62.66 ± 17.86	24/14	0/38	0/0/34/4	NA
TCGA	Illumina HiSeqV2	470	216	36.367	58.22 ± 15.73	290/180	103/364	77/140/171/23	NA
GSE91061	Illumina GPL9052	65	NA	81.07	NA	NA	NA	NA	CRPR:24/PD:24

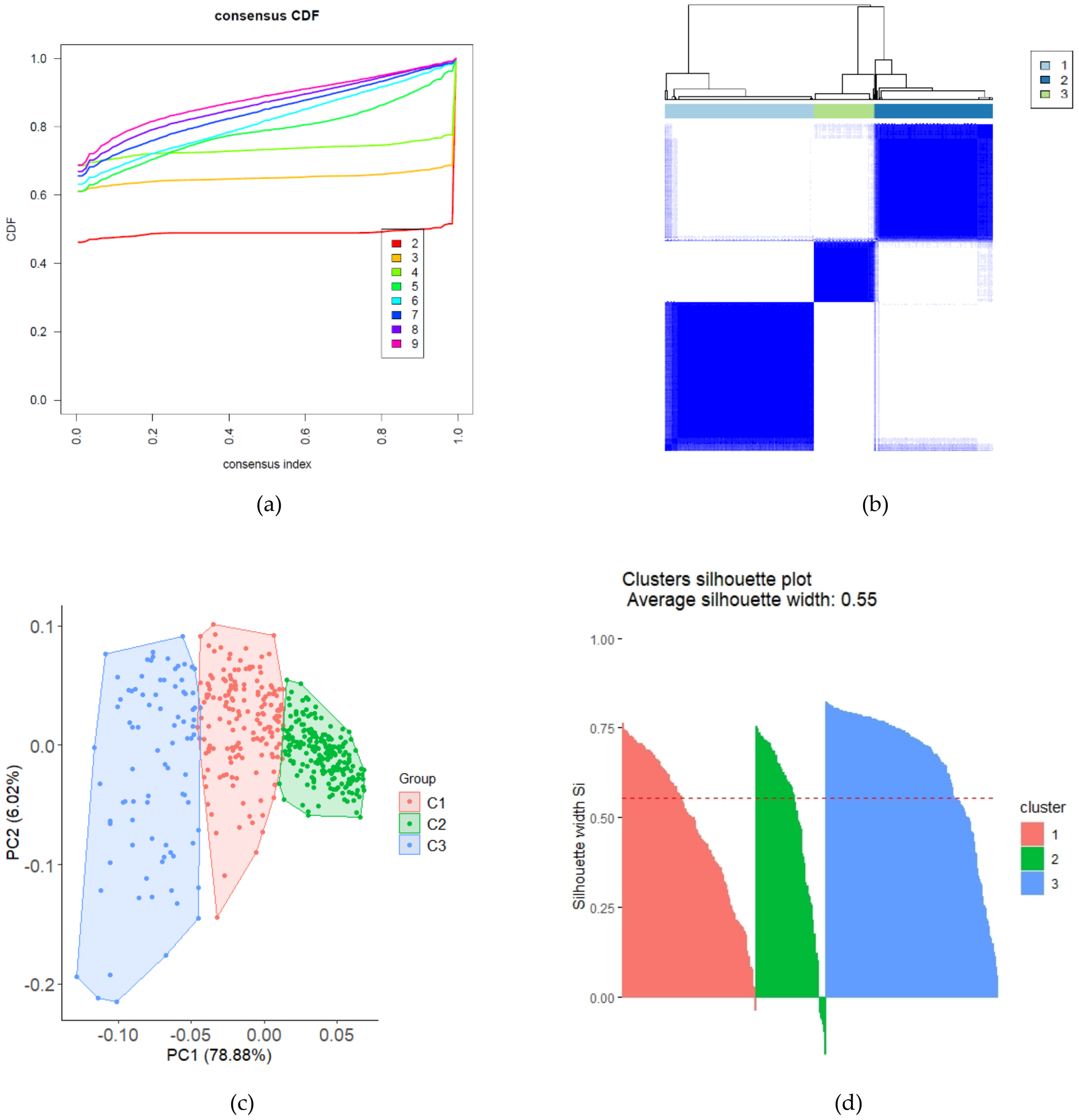

Figure S2. Validation of immune cluster numbers. (a) Cumulative distribution function (CDF) curve of the consistency score for different subtype numbers (k = 2–9); (b) The consensus score matrix for TCGA melanoma samples when k = 3 ;(c) Principal component analysis (PCA) of all samples based on ssGSEA scores; each point represents a sample; (d) Silhouette plot indicate the number of samples and average silhouette width for each cluster, the overall silhouette width represented at the bottom right of each chart. Higher average width indicates optimal number of cluster.

Table S2. Twenty eight immune related gene-sets.

Name	Ref	Gene
aDCs	*(J Exp Clin Cancer Res. 2018) PMID: 30594216*	CD83 LAMP3 CCL1
B cells	*(J Exp Clin Cancer Res. 2018) PMID: 30594216*	BACH2 BANK1 BLK BTLA CD79A CD79B FCRL1 FCRL3 HVCN1 RALGPS2
CCR	*(J Exp Clin Cancer Res. 2018) PMID: 30594216*	CCL16 TPO TGFBR2 CXCL2 CCL14 TGFBR3 IL11RA CCL11 IL4I1 IL33 CXCL12 CXCL10 BMPER BMP8A CXCL11 IL21R IL17B TNFRSF9 ILF2 CX3CR1 CCR8 TNFSF12 CSF3 TNFSF4 BMP3 CX3CL1 BMP5 CXCR2 TNFRSF10D BMP2 CXCL14 CCL28 CXCL3 BMP6 CCL21 CXCL9 CCL23 IL6 TNFRSF18 IL17RD IL17D IL27 CCL7 IL1R1 CXCR4 CXCR2P1 TGFB1I1 IFNGR1 IL9R IL1RAPL1 IL11 CSF1 IL20RA IL25 TNFRSF4 IL18 ILF3 CCL20 TNFRSF12A IL6ST CXCL13 IL12B TNFRSF8 IL6R BMPR2 IFNE IL1RAPL2 IL3RA BMP4 CCL24 TNFSF13B CCR4 IL2RA IL32 TNFRSF10C IL22RA1 BMPR1A CXCR5 CXCR3 IFNA8 IL17REL IFNB1 IFNAR1 TNFRSF1B CCL17 IFNL1 IL16 IL1RL1 ILK CCL25 ILDR2 CXCR1 IL36RN IL34 TGFB1 IFNG IL19 ILKAP BMP2K CCR10 ILDR1 EPO CCR7 IL17C IL23A CCR5 IL7 EPOR CCL13 IL2RG IL31RA TNFAIP6 IFNL2 BMP1 IL12RB1 TNFAIP8 IL4R TNFRSF6B TNFAIP8L1 TNFRSF10B IFNL3 CCL5 CXCL6 CXCL1 CCR3 TNFSF11 CSF1R IL21 IL1RAP IL12RB2 CCL1 IL17RA CCR1 IL1RN TNFRSF11B TNFRSF14 IL13 IL2RB BMP8B CCL2 IL24 IL18RAP TGFBI TNFSF10 TNFRSF11A CXCL5 IL5RA TNFSF9 IL1RL2 TNFRSF13C IL36G IL15RA TNFRSF21 CXCL8 IL22RA2 TNFAIP8L2 IL18R1 IFNLR1 CXCR6 CCL3L3 TNFRSF1A IL17RE IFNGR2 IL17RC TNFAIP8L3 ILVBL TGFBRAP1 CCL4L1 CSF2RA CCRN4L CCL26 TNFAIP1 CCRL2 IFNA10 TNFRSF17 IFNA13 IL20 IL18BP CCL3L1 TNFSF12-TNFSF13 IL5 IL23R IL26 TNF TGFA CSF2 IL1F10 CXCL17 TNFSF13 IFNA4 IL37 IL12A IL7R IFNA1 IL1A IL4 IL2 CCL22 CSF3R IL10 IFNK TGFB2 IL1R2 IL1B IL17F IL27RA IL15 TNFSF8 IL36B XCL1 CXCL16 TNFRSF19 IL3 CCL3 IFNA2 BMPR1B IFNA21 TNFSF18 CCL8 IL17RB TNFRSF25 IL22 IL10RB IFNAR2 CCL18 IFNA16 CSF2RB IL36A TNFAIP3 IL13RA2 IL13RA1 CCR9 TNFRSF10A IFNA7 IFNW1 XCL2 TNFSF14 CCR2 BMP15 BMP10 CCL15-CCL14 TGFBR1 IFNA5 BMP7 IFNA14 IL20RB IL10RA IFNA17 CCR6 TGFB3 CCL15 CCL4 CCL27 TNFRSF13B TNFAIP2 IL31 IL17A TNFSF15 CCL19 IFNA6 IL9
pDCs	*(J Exp Clin Cancer Res. 2018) PMID: 30594216*	CLEC4C CXCR3 GZMB IL3RA IRF7 IRF8 LILRA4 PHEX PLD4 PTCRA
T cell co-inhibition	*(J Exp Clin Cancer Res. 2018) PMID: 30594216*	BTLA C10orf54 CD160 CD244 CD274 CTLA4 HAVCR2 LAG3 LAIR1 TIGIT
T cell co-stimulation	*(J Exp Clin Cancer Res. 2018) PMID: 30594216*	CD2 CD226 CD27 CD28 CD40LG ICOS SLAMF1 TNFRSF18 TNFRSF25 TNFRSF4 TNFRSF8 TNFRSF9 TNFSF14
Th1 cells	*(J Exp Clin Cancer Res. 2018) PMID: 30594216*	IFNG TBX21 CTLA4 STAT4 CD38 IL12RB2 LTA CSF2
Th2 cells	*(J Exp Clin Cancer Res. 2018) PMID: 30594216*	PMCH LAIR2 SMAD2 CXCR6 GATA3 IL26

TIL	*(J Exp Clin Cancer Res. 2018) PMID: 30594216*	ITM2C CD38 THEMIS2 GLYR1 ICOS F5 TIGIT KLRD1 IRF4 PRKCQ FCRL5 SIRPG LPXN IL2RG CCL5 LCK TRAF3IP3 CD86 MAL LILRB1 DOK2 CD6 PAG1 LAX1 PLEK PIK3CD SLAMF1 XCL1 GPR171 XCL2 TBX21 CD2 CD53 KLHL6 SLAMF6 CD40 SIT1 TNFRSF4 CD79A CD247 LCP2 CD3D CD27 SH2D1A FYB ARHGAP30 ACAP1 CST7 CD3G IL2RB CD3E FCRL3 CORO1A ITK TCL1A CYBB CSF2RB IKZF1 NCF4 DOCK2 CCR2 PTPRC PLAC8 NCKAP1L IL7R 6-Sep CD28 STAT4 CD8A LY9 CD48 HCST PTPRCAP SASH3 ARHGAP25 LAT TRAT1 IL10RA PAX5 CCR7 DOCK11 PARVG SPNS1 CD52 HCLS1 ARHGAP9 GIMAP6 PRKCB MS4A1 GPR18 TBC1D10C GVINP1 P2RY8 EVI2B VAMP5 KLRK1 SELL MPEG1 MS4A6A ARHGAP15 MFNG GZMK SELPLG TARP GIMAP7 FAM65B INPP5D ITGA4 MZB1 GPSM3 STK10 CLEC2D IL16 NLRC3 GIMAP5 GIMAP4 IFFO1 CFH PVRIG CFHR1
Treg	*(J Exp Clin Cancer Res. 2018) PMID: 30594216*	IL12RB2 TMPRSS6 CTSC LAPTM4B TFRC RNF145 NETO2 ADAT2 CHST2 CTLA4 NFE2L3 LIMA1 IL1R2 ICOS HSDL2 HTATIP2 FKBP1A TIGIT CCR8 LTA SLC35F2 IL21R AHCYL1 SOCS2 ETV7 BCL2L1 RRAGB ACSL4 CHRNA6 BATF LAX1 ADPRH TNFRSF4 ANKRD10 CD274 CASP1 LY75 NPTN SSTR3 GRSF1 CSF2RB TMEM184C NDFIP2 ZBTB38 ERI1 TRAF3 NAB1 HS3ST3B1 LAYN JAK1 VDR LEPROT GCNT1 PTPRJ IKZF2 CSF1 ENTPD1 TNFRSF18 METTL7A KSR1 SSH1 CADM1 IL1R1 ACP5 CHST7 THADA CD177 NFAT5 ZNF282 MAGEH1
Inflamma-tion-promoting	*(J Exp Clin Cancer Res. 2018) PMID: 30594216*	CCL5 CD19 CD8B CXCL10 CXCL13 CXCL9 GNLY GZMB IFNG IL12A IL12B IRF1 PRF1 STAT1 TBX21
Activated CD8 T cell	*(Immunity 2013) PMID: 24138885*	ADRM1 AHSA1 C1GALT1C1 CCT6B CD37 CD3D CD3E CD3G CD69 CD8A CETN3 CSE1L GEMIN6 GNLY GPT2 GZMA GZMH GZMK IL2RB LCK MPZL1 NKG7 PIK3IP1 PTRH2 TIMM13 ZAP70
TNFa_speed	*SPEED database PMID:20494976*	ACSL3 AGTR1 ALAS1 AMPD3 BDKRB1 BHLHE41 BID C13orf31 C15orf48 CDKN2B CH25H CHST15 CHST2 COL1A2 COL3A1 DAB2 DCUN1D3 DENND3 EIF1B ESPL1 FAS FGF2 FZD10 FZD7 GCLM GFPT2 GNG2 HIVEP2 HMGCS1 HOXA9 HSD11B1 ID4 IDH3A IFNAR2 IFNGR2 IL17RB IRAK2 KIAA1949 KIF1B KLF9 LITAF 44258 MFHAS1 MMP1 MMP3 MSC MT1F MT1P2 MT1X NAV2 NCOA7 NFKBIE NINJ1 NMB OSR1 PCDH18 PGM2L1 PHF17 PLK2 PLK3 PPP1R15B PPP1R3B PTGES PTPN1 RAP2C RASSF5 RNF103 RRAD RUNX1T1 SAMD4A SERPINB8 SIRPA SLC19A2 SLC25A37 SLC2A1 SLC2A6 SLC39A14 SLC39A8 SLC7A2 SPSB1 SRGN TJP2 TMEM171 TNC TNFRSF10B TP53INP2 TRIB2 TRIM47 TRRAP TSC22D1 TSC22D3 TYMP UBTD2 UGP2 USP12 UXS1 XBP1 ZC3H12C ZNF267 ZNF295 ZNF395
IFNG	*(NatureGen2014 PMID:25151356)*	APOL2 APOL6 ARNTL ATP6V1C1 BATF3 BCL6 BTN3A1 BTN3A2 BTN3A3 C19orf66 C1orf38 CD2AP CD47 CTRL CXCL10 CXCL11 CXCL9 DDX58 DNPEP ETV1 FOSL2 FST GBP1 GCH1 GPD2 HERC6 IDO1 IFI30 IFI35 IFI44 IFI44L IFI6 IFIT2 IFIT3 IFIT5 IFITM1 IL15 IRF1 IRF8 JAK2 KCTD14 LAP3 LPIN1 MX1 NHEJ1 NNMT OAS2 PCDH7 PION PLSCR1 PMAIP1 PPA1 PRRG1 PSMB9 RARRES3 RNF114 RNF19B RSAD2 RTP4 SBNO2 SCYL3 SECTM1 SETBP1 SLC15A3 SLC25A28 SP100 SP110 STAT1 STAT2 TAP1 TAP2 TESK2 TNFAIP2 TRAFD1 TRIB1 TRIB2 TRIM14 TRIM21 UBE2L6 ZC3HAV1
TNFA	*(NatureGen2014 PMID:25151356)*	ACSL3 ALCAM APOL2 BIRC2 BTN3A3 C8orf4 CCL2 CD47 CDV3 CFLAR CXCL1 CXCL10 CXCL5 CYLD DDX58 DNAJA1 FZR1 GATA6 HERC6 HIVEP2 HNF4G HRH1 IL15 IL8 ISG20 KIAA0247 KIAA1609 KIAA1609 LYRM1 MMP12 MREG MT1X MYD88

		NECAP2 NFE2L3 NINJ1 NKX3-1 OAS2 OGFR OTUD4 PARP12 PHF11 PIK3CD PSTPIP2 SLC2A6 SLIT2 SMOX SP100 SRPK1 TNFAIP2 TNFAIP3 TNFAIP6 TNFRSF11B TRAF1 TRIM21
IL-1_speed	*(SPEED database PMID:20494976)*	AKR1C2 APOL3 C19orf6 CDV3 CSF1 CX3CL1 DUSP8 EFNA1 FEZ2 FJX1 IL18R1 ITGB5 JUND LTB MARCKSL1 MSX1 NNMT PI3 PPIF SELE SMAD3 SPRR2B UPP1
T cell_infilt-1	*(Spranger Nature2015) PMID:25970248)*	CD8A CCL2 CCL3 CCL4 CXCL9 CXCL10 ICOS GZMK IRF1 HLA-DMA HLA-DMB HLA-DOA HLA-DOB
T cell_infilt-2	*(Harlin CancerRes2009) PMID:19293190)*	CD8B CCL4 CCL5 CXCL9 XCL2 CXCL10 CXCL11 CXCL13 CCL2 CCL19 CCL21
BIOCARTA_IL12_PATHWAY	*Msigdb(http://www.gsea-msigdb.org/gsea/msigdb/index.jsp)*	CCR5 CCR5 CD247 CD247 CD3D CD3D CD3E CD3G CXCR3 CXCR3 ETV5 IFNG IL12RB1 IL12RB1 IL12RB2 IL12RB2 IL12RB2 IL12RB2 IL18 IL18 IL18R1 JAK2 JUN MAP2K6 MAPK14 MAPK14 MAPK14 MAPK14 MAPK8 MAPK8 MAPK8 STAT4 STAT4 TYK2
iDC	*(Immunity 2013) PMID: 24138885*	ABCG2 BLVRB CARD9 CD1A CD1B CD1C CD1E CH25H CLEC10A CSF1R CTNS F13A1 FABP4 FZD2 GSTT1 GUCA1A HS3ST2 LMAN2L MMP12 MS4A6A NUDT9 PDXK PPARG PREP RAP1GAP SLC26A6 SLC7A8 SYT17 TACSTD2 TM7SF4 VASH1
Effector memory CD4+ T cells	*(Genome Biol (PMID:25853550)*	BMI1 CASP3 CNOT10 COPB2 DARS EXOSC9 EZH2 GDE1 IFT74 KLF5 NDUFB9 NUP205 SHCBP1 XRCC6
NKcells	*(Immunity 2013) PMID: 24138885*	AKT3 AXL BCL2 BST2 CDC5L CDH2 CRTAM CSF2RA CTSZ CXCL1 CYTH1 DAXX DGKH DLL4 DPYD ERBB3 F11R FAM27A FAM49A FASLG FCGR1A FGF18 FN1 FSTL1 FUCA1 FUT5 FZR1 GAGE2 GBP3 GLS2 GRB2 IGFBP5 KANK2 LDB3 LST1
CD8+ T cells	*(Genome Biol (PMID:25853550)*	ADRM1 AHSA1 C1GALT1C1 CCT5 CCT6B CETN3 CSE1L EIF2S1 GAL GEMIN6 GPT2 KIAA0101 MND1 MPZL1 MRPS16 PCNA PTRH2 RFC5 SPC25 TIMM13 TIMM8B TK1 TUBB TXNDC17
Activated B cell	*(Immunity 2013) PMID: 24138885*	ADAM28 CD180 CD79B BLK CD19 MS4A1 TNFRSF17 GNG7 MICAL3 SPIB HLA-DOB PNOC FCRL2 BACH2 CR2 TCL1A AKNA ARHGAP25 CCL21 CD27 CD38 CLEC17A CLEC9A CLECL1
T.cells.CD8.naive	*(Genome Biol) PMID:31892342*	CCR7 LEF1 SELL TCF7 S1PR1 CD27 CD28
DC	*(Genome Biol (PMID:25853550)*	C1QC CCDC88A CCL13 CCL3L1 CCL3L3 CD300E CD86 CLEC1A CLEC4C CLEC5A CLIC2 CSF2RA FAM49A FCGR1A FN1 FSTL1 GPR109B GPX3 HLA-DQA2 INHBA LGMN NGFR PDGFRL PDPN PRKAR2B SIGLEC1 SIGLEC5 SLAMF9 STAB1 THBD TNFAIP2 TTYH2 UBD VCAM1
Cytotoxic cells	*(Immunity 2013) PMID: 24138885*	APBA2 APOL3 CTSW DUSP2 GNLY GZMA GZMH KLRB1 KLRD1 KLRF1 KLRK1 NKG7 RORA RUNX3 SIGIRR WHAMMP3 ZBTB16
cytotoxic memory CD4 T cell	*(Genes Immun 2005) PMID: 15789058*	ABCB4 ABCB9 ACAP1 ACHE ACP5 ADAM28 ADAMDEC1 ADAMTS3 ADRB2 AIF1 AIM2 ALOX15 ALOX5 AMPD1 ANGPT4 ANKRD55 APOBEC3A APOBEC3G APOL3 APOL6 AQP9 ARHGAP22 ARRB1 ASGR1 ASGR2 ATHL1 ATP8B4 ATXN8OS AZU1 BACH2 BANK1 BARX2 BCL11B BCL2A1 BCL7A BEND5 BFSP1 BHLHE41

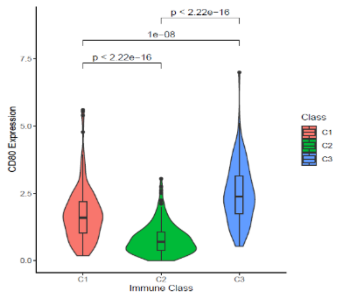

(a)

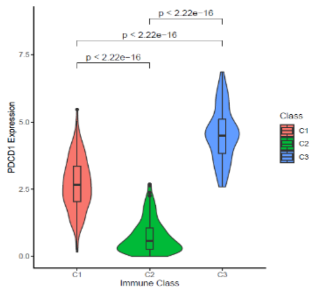

(b)

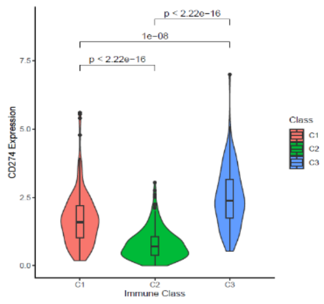

(c)

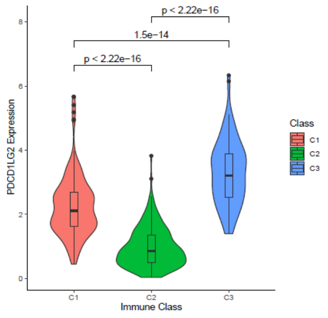

(d)

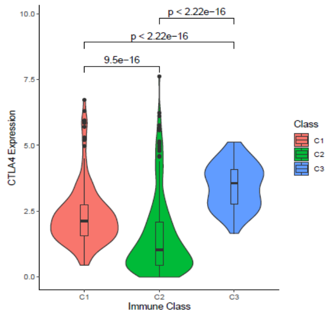

(e)

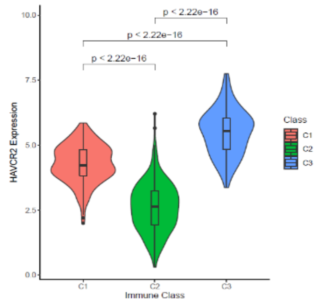

(f)

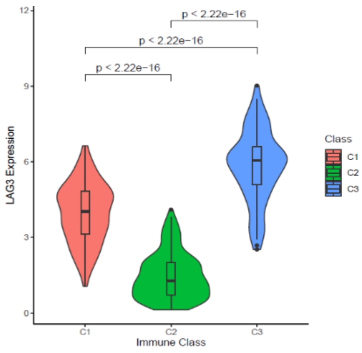

(g)

Figure S3. Differential expression of the immune checkpoint genes. The expression of CD80, PDCD1, CD274, PDCD1LG2, CTLA4, HAVCR2, and LAG3 (a-g), between all three subtypes evaluated by Mann–Whitney U-test; bars indicate medians. P < 0.001.

Table S3. Immune evasion mechanaims

Category	Mechanism	Sub Mechanism	Gene set	PMID
**Tumor extrinsic**	Immunosuppressive TME	Hypoxia	HALLMARK_HYPOXIA	33305856
		TGFB mediated immune evasion	HALLMARK_TGF_BETA_Signaling	33291370
		DC activation	Activated Dendritic cell	30594216
	Aberrant Vasculature	Angiogenesis	HALLMARK_ANGIOGENESIS	30183339
**Tumor intrinsic**	Oncogene-related Pathway	Lack of PTEN	BIOCARTA_PTEN_Pathway	32327707
		NOTCH_Signaling	HALLMARK_NOTCH_ Signaling	30061899
		WNT-beta catenin activation	HALLMARK_WNT/β_Catenin_ Signaling	25970248
		PI3K_AKT_MTOR_Signaling	HALLMARK_PI3K_AKT_MTOR_ Signaling	31874279
		Dysregulated MAPK Pathway	BIOCARTA_MAPK_Pathway	31874279
		MYC oncogene activation	HALLMARK_MYC_TARGETS	34508258
	Lack of tumor antigens	Cancer cell antigen presenting	KEGG_Antigen processing and presentation	26748421
	Metabolic competition	Glycolysis	HALLMARK_ Glycolysis	28400131
		Oxidative Phosphorylation	HALLMARK_ Oxidative Phosphorylation	22568930
		Alterations in fatty acid metabolism	HALLMARK_Fatty_Acid_Metabolism	31819192
	Defective DNA repair	Defective DNA repair	KEGG Mismatch repair	30665488
			HALLMARK_DNA_Repair	30665488
	lose of cell polarity	Epithelial–mesenchymal transition	HALLMARK_EMT	31605776

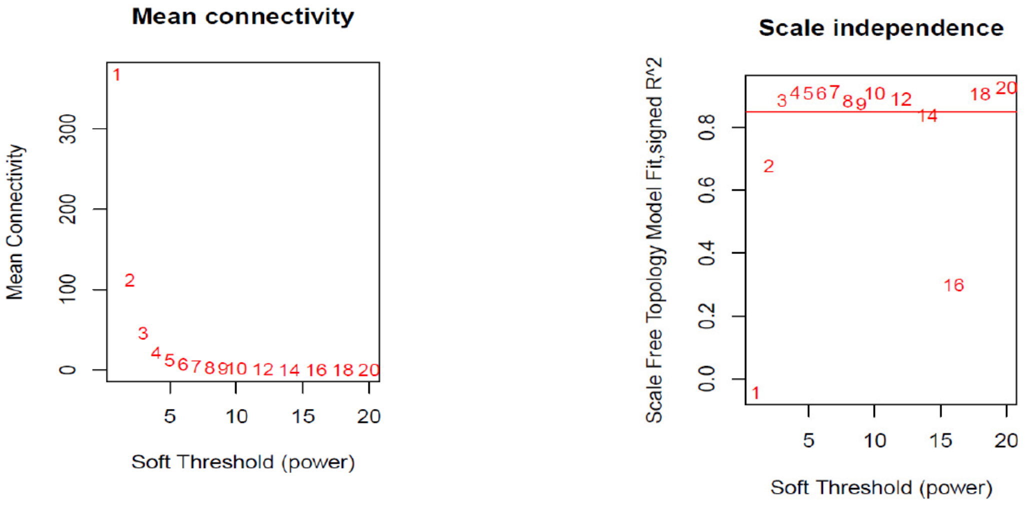

(a) (b)

Figure S4. Network topology for different soft-thresholding powers. (a) The scale-free topology index as a function of the soft-thresholding power; (b) the mean connectivity as a function of the soft-thresholding power.

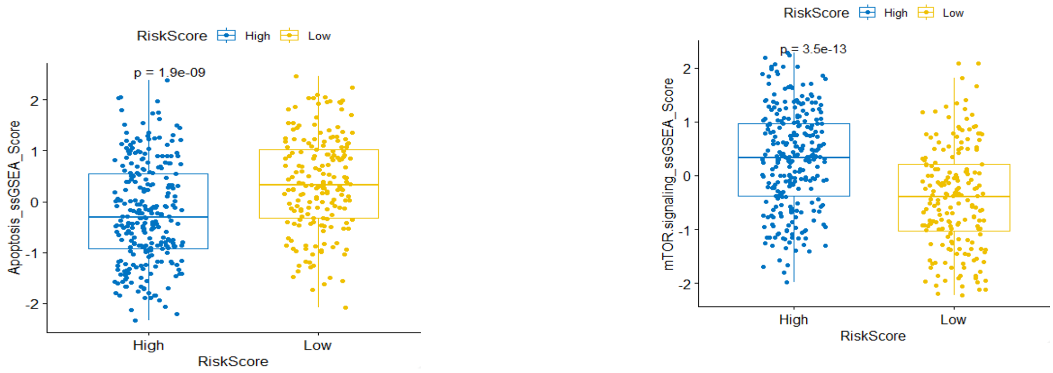

(a) (b)

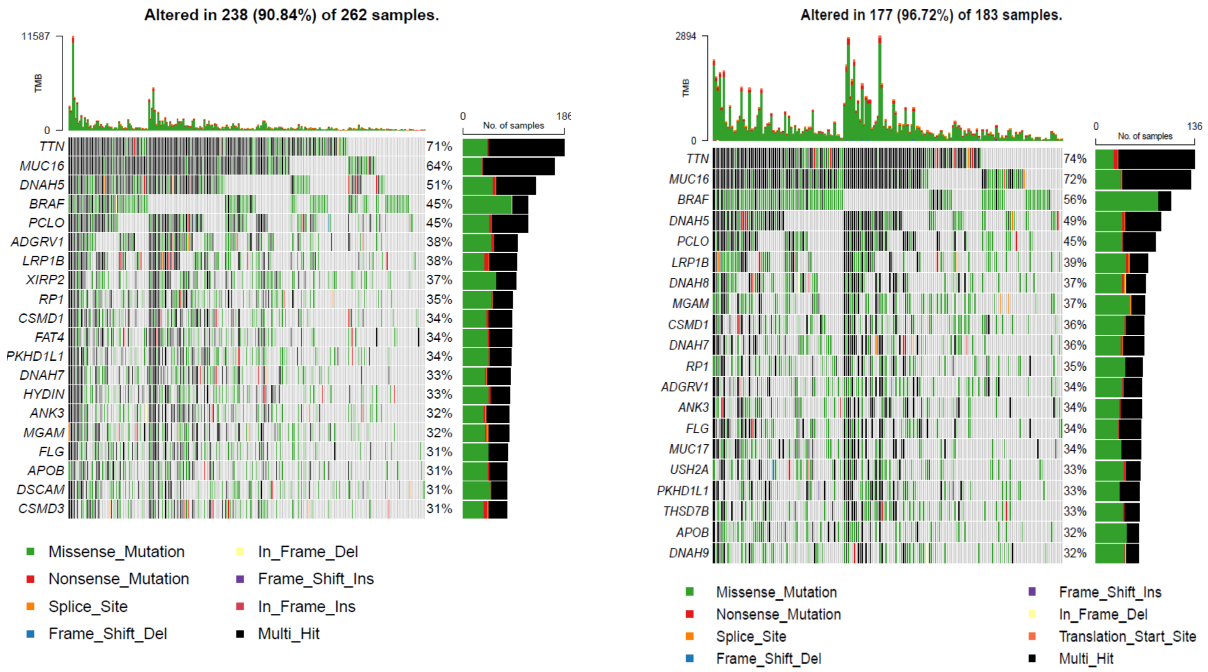

(c) (d)

Figure S5. (a,b) Boxplot of ssGSEA normalized scores of the two representative pathways in the high and low risk score groups. In each box, the central line represents the median value, and the bounds represent the 25th and 75th percentiles; (c) mutational status of high -risk group; (d) mutational status of low -risk group.

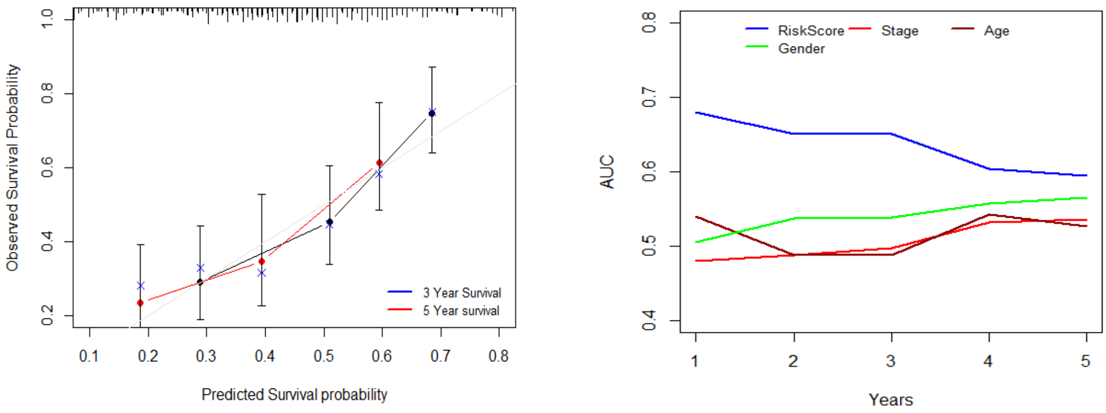

(a) (b)

Figure S6. (a) calibration plot of GSE65904 nomogram; (b) AUC curves of risk score -related gene signature nomogram compared with stage, age and and gender in the GSE65904 (1 to 5-years).
